# The Croatian psycholinguistic database: Estimates for 6000 nouns, verbs, adjectives and adverbs

**DOI:** 10.3758/s13428-020-01533-x

**Published:** 2021-04-26

**Authors:** Anita Peti-Stantić, Maja Anđel, Vedrana Gnjidić, Gordana Keresteš, Nikola Ljubešić, Irina Masnikosa, Mirjana Tonković, Jelena Tušek, Jana Willer-Gold, Mateusz-Milan Stanojević

**Affiliations:** 1 Faculty of Humanities and Social Sciences, University of Zagreb, Ivana Lučića 3, 10000 Zagreb, Croatia; 2 Department of South Slavic Languages and Literatures, Zagreb, Croatia; 3 Department for German Language and Literature, Zagreb, Croatia; 4 Department of Psychology, Zagreb, Croatia; 5 Department of Information Sciences, Zagreb, Croatia; 6 Department of Knowledge Technologies, Jožef Stefan Institute, 1000 Ljubljana, Slovenia; 7 Division of Psychology and Language Sciences, University College London, London, WC1N 1PF UK; 8 Department of English, Zagreb, Croatia

**Keywords:** Croatian psycholinguistic database, Concreteness, Imageability, Age of acquisition, Subjective frequency, Computational modeling

## Abstract

Psycholinguistic databases containing ratings of concreteness, imageability, age of acquisition, and subjective frequency are used in psycholinguistic and neurolinguistic studies which require words as stimuli. Linguistic characteristics (e.g. word length, corpus frequency) are frequently coded, but word class is seldom systematically treated, although there are indications of its significance for imageability and concreteness. This paper presents the Croatian Psycholinguistic Database (CPD; available at: 10.17234/megahr.2019.hpb), containing 6000 Croatian nouns, verbs, adjectives and adverbs, rated for concreteness, imageability, age of acquisition, and subjective frequency. Moreover, we present computationally obtained extrapolations of concreteness and imageability to the remainder of the Croatian lexicon (available at: https://github.com/megahr/lexicon/blob/master/predictions/hr_c_i.predictions.txt). In the two studies presented here, we explore the significance of word class for concreteness and imageability in human and computationally obtained ratings. The observed correlations in the CPD indicate correspondences between psycholinguistic measures expected from the literature. Word classes exhibit differences in subjective frequency, age of acquisition, concreteness and imageability, with significant differences between nouns, verbs, adjectives and adverbs. In the computational study which focused on concreteness and imageability, concreteness obtained higher correlations with human ratings than imageability, and the system underpredicted the concreteness of nouns, and overpredicted the concreteness of adjectives and adverbs. Overall, this suggests that word class contains schematic conceptual and distributional information. Schematic conceptual content seems to be more significant in human ratings of concreteness and less significant in computationally obtained ratings, where distributional information seems to play a more significant role. This suggests that word class differences should be theoretically explored.

## Introduction

Psycholinguistic databases containing human ratings of characteristics such as concreteness, imageability, age of acquisition, and subjective frequency are routinely used in a variety of psycholinguistic and neurolinguistic studies which require words as stimuli (for a review of such studies, see Vigliocco et al., 2011). Various linguistic characteristics (e.g. word length and corpus frequency) are also frequently coded in databases. However, word class has rarely been systematically treated, although it has been found that words belonging to different word classes exhibit differences in imageability (Bird et al., 2001; Simonsen et al., 2013) and concreteness (Peti-Stantić et al., 2018). This is not surprising if word class is considered meaningful, i.e. as providing semantic, conceptual and distributional information (Langacker, 2008).

Extrapolations of word characteristics using computational modeling (e.g. Buechel et al., 2020) are another area where semantic and distributional information is crucial. Collecting human ratings is a resource-intensive process, and for less researched languages such as Croatian, obtaining high-quality computationally generated scores for the untested part of the lexicon may be a cost-effective alternative. However, for this to happen, we need to be relatively certain that no semantic artifacts are introduced in the extrapolated ratings (Mandera et al., 2015). For instance, whereas extrapolated concreteness ratings for English consistently exhibit high correlations with human ratings across studies (Hollis et al., 2017; Ljubešić et al., 2018), correlations for some extrapolated affective variables have been somewhat lower for English and some other languages (Buechel et al., 2020; Hollis et al., 2017). In addition to the word characteristic in question, the reasons behind this may lie in the technique used, i.e. how semantic space is reconstructed from distributional information, and which machine-learning or statistical method is used (Mandera et al., 2015). Similarly, it is reasonable to assume that semantic and distributional data coded in word class may play a role in the quality of the extrapolation. Croatian is a good test case in this sense, as it has a rich morphology which largely signals word class (unlike, for instance, English). Moreover, morphologically it is a relatively typical representative of the group of Slavic languages, which seems underrepresented in the available literature, but accounts for some 315 million speakers (Ivanov & Brown, 2020).

In this paper, we present the Croatian Psycholinguistic Database (CPD; Peti-Stantić et al., 2019, available at 10.17234/megahr.2019.hpb) with 6000 Croatian nouns, verbs, adjectives and adverbs selected in a systematic way. The database includes values for word length, word class, animacy and corpus frequency, as well as ratings of concreteness, imageability, age of acquisition (AoA), and subjective frequency. We then focus on a computational model that extends the ratings of concreteness and imageability to the remaining 100,000 words in the Croatian lexicon for which the ratings were not collected in the CPD. The computationally obtained ratings are freely available at https://github.com/megahr/lexicon/blob/master/predictions/hr_c_i.predictions.txt. In the two studies we conceive of word class as a “shorthand” for conceptual and distributional characteristics, which leads to an account where concreteness and imageability are ultimately considered different measures, with concreteness more clearly related to distributional characteristics. In the remainder of the introduction, we focus on the word characteristics in the CPD, other databases and their limitations, extrapolating word characteristics and the theoretical significance of word classes for concreteness and imageability effects.

### Word characteristics

Concreteness is the degree to which a word refers to an entity that can be experienced by the senses (Paivio et al., 1968). A facilitatory effect of concrete words, dubbed the concreteness effect, was reported in a number of experimental tasks and paradigms, including word naming (De Groot, 1989), lexical decision (Binder et al., 2005), learning new vocabulary (De Groot & Keijzer, 2000), and free recall (Fliessbach et al., 2006; Romani et al., 2008). Reaction time studies and electrophysiological measures (ERPs) also confirm facilitated processing of concrete words (e.g. Barber et al., 2013; Kanske & Kotz, 2007; Kounios & Holcomb, 1994; Schwanenflugel et al., 1992; West & Holcomb, 2000). However, a reverse effect was recently reported both in experiments with patients (Yi et al., 2007) and with healthy participants (Barber et al., 2013; Kousta et al., 2011).

Imageability refers to how easily and quickly a word evokes a mental image in different modalities (Paivio et al., 1968). Effects of imageability were first reported in patients with aphasia (Martin et al., 1996; Nickels & Howard, 1995) and deep dyslexia (Plaut & Shallice, 1993). Given that concreteness and imageability exhibit a high correlation (Paivio et al., 1968), some researchers use the two categories interchangeably (e.g. Reilly & Kean, 2007). However, more recent work shows that highly abstract words which are highly imageable may be affectively saturated (Dellantonio et al., 2014; Kousta et al., 2011).

Norms for age of acquisition (AoA) are often based on subjective ratings which have been found to correlate with objective measures of AoA (Morrison et al., 1997). Many studies show an effect of AoA in picture and word naming, object recognition and lexical decision tasks (see Juhasz, 2005; Łuniewska et al., 2016, p. 1156–1157), as well as in retrieving meanings of words (Marful et al., 2016; Navarrete et al., 2015).

Subjective frequency refers to the participants’ assessment of how frequently they encounter a word, which has been suggested as more straightforward for participants than rating subjective familiarity (Balota et al., 2001). Subjective frequency was found to correlate with corpus-based, objective frequency counts (Brysbaert & New, 2009). Both objective and subjective frequency may impact linguistic processing and need to be controlled so as not to confound the results of psycholinguistic experiments. As has been previously noted (e.g. Balota et al., 2001; Mayberry et al., 2014), lexical frequency has been used to model the acquisition of the mental lexicon as well as its organization and processing (Bock & Griffin, 2000; Dahan et al., 2001; Dell, 1990; Gardner et al., 1987; Juhasz et al., 2019). There is still a debate as to the importance of objective vs. subjective frequency: whereas some researchers believe that the importance of subjective frequency is overemphasized in psycholinguistics (Brysbaert & Cortese, 2011), others show its significance (Kuperman & Van Dyke, 2013).

Word length, animacy and word class are also routinely controlled in psycholinguistic research. Word length influences a variety of cognitive processes, including lexical access and memory (see Barton et al., 2014, for a review). Animacy is the difference between animate and inanimate entities, which influences semantic processing (see Radanović et al., 2016, for a review). Words belonging to different word classes have been found to be related to different imageability ratings (e.g. Bird et al., 2001; Simonsen et al., 2013), and given the correlation between imageability and concreteness, the same is reasonable to expect for concreteness, although the data is scarce.

### Databases, standards and limitations

Human ratings of these and other characteristics appear in databases for a number of languages, for instance English (Bird et al., 2001; Brysbaert et al., 2014b; Coltheart, 1981; Paivio et al., 1968), Spanish (Duchon et al., 2013; Guasch et al., 2016), Italian (Della Rosa et al., 2010; Montefinese et al., 2019; Rofes et al., 2018), French (Desrochers & Thompson, 2009), Dutch (Brysbaert et al., 2014a), Portuguese (Soares et al., 2017), Polish (Imbir, 2016) and Chinese (Yee, 2017). Most of them are freely available, but some are only available as lists rather than downloadable datasets. They differ significantly in the number of words (from several hundred to tens of thousands), word selection procedures, features that are rated, and the comprehensiveness of the available data (e.g. whether individual data points are available for each word). Concreteness and imageability ratings are largely provided for nouns, although databases now include other word classes (Bird et al., 2001; Duchon et al., 2013; Guasch et al., 2016; Imbir, 2016; Simonsen et al., 2013; Soares et al., 2017). Still, word class differences are rarely explored.

As far as Croatian is concerned, the previously compiled Croatian Lexical Database (Kuvač Kraljević & Olujić, 2018) is a meta-database, with imageability, subjective frequency, concreteness, familiarity, AoA, word class and word length ratings for 2869 words collected from seven different studies between 2007 and 2019. A total of 600 words in the database were rated by at least 23 raters and are thus a valuable resource. The remaining 2269 words were rated by three or fewer raters and should be approached with caution. Another database available for Croatian (Ćoso et al., 2019) contains ratings of 3022 Croatian words for valence, arousal and concreteness. The database is freely available and includes verbs, nouns and adjectives, but word class has not been coded in the database. As is evident from the instructions used in the study, concreteness of a word was defined as “the degree of specificity of its content”, i.e. the number of referents that it can have, rather than by using the prevalent definition of the availability of sensory information. Therefore, the resulting concreteness ratings are not directly comparable to other databases.

### Extrapolating word characteristics

As has already been said, constructing large-scale databases which include much of the lexicon is a resource-intensive process, and is difficult to do for a relatively understudied language such as Croatian, as the comparative paucity of published data on Croatian shows. Even the CPD, with its 6000 words, covers only a small part of the lexicon (cf. the large databases such as Brysbaert et al., 2014a, 2014b, and , for English and Dutch with 40,000 and 30,000 words, respectively). Therefore, regression and machine learning techniques (cf. Crossley et al., 2013, for the former and Hollis et al., 2017, for the latter) have been used to extend human ratings to the remainder of the lexicon within a single language or to other languages for psycholinguistic variables such as concreteness and imageability (Ljubešić et al., 2018; Thompson & Lupyan, 2018) and affective variables (valence, arousal and dominance [Buechel et al., 2020; Hollis et al., 2017; Recchia & Louwerse, 2015]).

In machine learning studies, word embeddings are often used. Word embeddings are numerical representations of words, usually in the form of n-dimensional vectors. Considering that vectors can mathematically be represented as positions in an n-dimensional Cartesian space, every word-related vector assigns a unique “address” in space to every word. Word vectors are calculated using the positions of words within large language corpora, based on their neighboring words and in line with observations first made by Harris (1954), and later popularized by Firth’s (1957, p. 11) adage “You shall know a word by the company it keeps.” Different words that occur many times in similar contexts within a corpus, such as *nectarines* and *peaches* (in “Nectarines are stone fruits” and “Peaches are stone fruits”), will thus have similar vector values and appear as close in the spatial representation of that particular corpus. What is even more interesting for our work, the N values of the resulting n-dimensional vectors were proven to encode various linguistic properties of words.

There have been warnings that extrapolation may create semantic artifacts absent from the human ratings for AoA, concreteness and affective variables, even if correlations are high (Mandera et al., 2015). Models using word embedding dimensions as explanatory variables limit this, particularly for concreteness and less so for the affective variables (Hollis et al., 2017), although perfect correlations have not been achieved. Given that concreteness and imageability are highly correlated, we will explore what happens with the model using word embedding dimensions as explanatory variables for these two measures, particularly with relation to word class.

### Theoretical significance of word class for concreteness and imageability

Human ratings of word characteristics and computational extrapolation data are closely related to word class data: the former increasingly include it, as noted above, and the latter are based on distributional characteristics, which also means that they are necessarily sensitive to different word classes. Word classes have rarely been included as part of theories explaining concreteness and imageability effects, although there are empirical and theoretical reasons for their inclusion. For instance, Simonsen et al. (2013) found that the imageability of Norwegian nouns and verbs differs significantly, with nouns being more imageable than verbs. From the neurocognitive perspective, words belonging to different word classes are processed differently (Lee & Federmeier, 2008), even though the data are sometimes ambiguous for languages such as English, where additional disambiguation cues are required to isolate the effects of word class. Language acquisition studies indicate that English-speaking children learn nouns first, whereas function words are learned only after the rudiments of syntax with multi-word utterances are set in place (Bates et al., 1994; Caselli et al., 1995). Moreover, there is reason to believe that, at least in morphologically rich languages such as Croatian, typical semantic clues which are part-and-parcel of word class information (nouns typically refer to objects and verbs to relations) have consequences for their neural representation and processing (Vigliocco et al., 2011).

This last view hinges on treating word class and other grammatical information as meaningful, which is in line with cognitive linguistic treatments (Langacker, 2008). More specifically, grammar provides semantic, conceptual and distributional information, which constitutes every lexical and multi-word item, but is more schematic than typical lexical information (Langacker, 1987). For instance, whereas the noun *book* refers to an object, the verb *to book* refers to an action. More generally, nouns typically denote conceptually independent entities defined in the spatial domain, while verbs denote conceptually dependent (i.e. relational) entities defined in time and being diffuse in space (Langacker, 1987). Adjectives and adverbs are atemporal relations, which means that they refer to other entities, but not in the domain of time. Adjectives refer to the domain of quality and require the separation of the quality from the entity that has the quality. For example, describing an object as *a wooden table* requires separating the material (wood) from the object (table). Adverbs are more varied than adjectives, and may refer to the spatial or temporal domain, as well as various other circumstances (cause, effect, etc.).

This information is also related to grammatical valence as conceptually defined by Croft (1991). Grammatical valence in this sense refers to the extent to which a word requires other elements to be fully grammatically realized. Nouns have a grammatical valence of 0, which means that they do not need any other words to appear with them. Verbs have a valence of at least 1, which means that they need at least one other word (typically a noun or a pronoun) to combine with them (for instance, the verb *run* requires the addition of an entity that is doing the running). Finally, adjectives and adverbs have the grammatical valence of exactly 1, because the way they describe qualities and circumstances requires a noun/verb, respectively, whose quality/circumstance is realized. In this sense, adverbs are further removed from adjectives, because they work with verbs which have their separate grammatical valence of at least 1.

These explanations shed new light on the three leading models that have been developed to account for the processing differences of concrete and high-imageable words on the one hand, and abstract and low-imageable words on the other: the context availability theory (Schwanenflugel & Shoben, 1983), the dual-coding theory (Paivio, 1986) and grounding theories (e.g. Barsalou, 2008). In essence, all three theories, alongside experiential knowledge, posit a distinctive role of the verbal/linguistic knowledge in achieving concreteness effects. In the context availability theory, contextual knowledge, i.e. verbal information, is crucial: concrete words have more numerous and stronger associations to contextual knowledge, while abstract words have fewer and weaker associations. The dual-coding theory claims that concrete words are represented perceptually and verbally, whereas abstract words are primarily represented verbally, lacking sensory referents (Paivio, 2010). In grounding theories, semantic knowledge is not considered amodal, but as based on the brain’s modal system for “perception, action and introspection” (Barsalou, 2008, p. 619). Concrete and highly imageable words are understood directly, based on our physical interaction with them and our perceptual experience of them. Understanding abstract concepts is variably explained (for a review see Pecher et al., 2011), but more recently linguistic, and particularly affective, information has been brought to bear (Hinojosa et al., 2020; Kousta et al., 2011; Vigliocco et al., 2014). These accounts claim that experiential and linguistic information play a role in understanding all concepts (both abstract and concrete), with affective and linguistic information weighing in more on abstract concepts, and sensorimotor information on concrete concepts. Given the significance of linguistic information posited in all three theories, word class should have an effect on concreteness and imageability, because typical word class information is a shorthand for semantic and distributional clues, as shown above.

## The current study

In what follows we provide a comprehensive and unified database with 6000 Croatian nouns, verbs, adjectives and adverbs, selected in a systematic way, which includes word length, word class, animacy and corpus frequency as well as ratings of concreteness, imageability, AoA, and subjective frequency. We present the behavioral study in detail, as we consider the resulting ratings to be a starting point for psycholinguistic studies to come. We use the results of the study, particularly the relation between concreteness, imageability and word class, as the baseline for the computational study, which we present next. In it, we test a predictive computational model extrapolating concreteness and imageability to the remainder of the Croatian lexicon, and compare it with English data. Based on the results of the two studies, we discuss the significance of word class in explaining concreteness and imageability effects in human ratings and machine learning as well as the significance of different types of evidence for machine learning.

## Study 1: Human ratings

### Method

#### Participants

A total of 3630 questionnaires were completed by native speakers of Croatian, students at the University of Zagreb (Faculty of Humanities and Social Sciences, Faculty of Teacher Education) and the University of Rijeka (Faculty of Humanities and Social Sciences). No monetary compensation was offered, but some students were awarded class credit for their participation. The mean age of the participants was 21.18 (SD = 2.61), and their age range was 18–50. There were 78.45% females and 21.55% of males. Most participants (69.32%) reported speaking two or more foreign languages (mean number of foreign languages spoken 2.25, SD = 1.03). Most participants (79.67%) reported that they spent between 1 and 4 hours reading per day on average. Every word was rated by an average of 30 participants (see Table 1 in the Appendix for the details).

#### Materials

The ratings were collected in two rounds of 3000 words each. For the first round, words were excerpted from hrWaC, a 1.2 billion web corpus of Croatian (Ljubešić & Klubička, 2016) by combining the hrLex inflectional lexicon (Ljubešić, 2019) with objective word frequencies from hrWac. Only words with a raw frequency of over 3000 were excerpted. Out of the resulting 7695 nouns, 2849 verbs and 3124 adjectives, 1000 words per each word class were randomly selected for testing. In the second round, an additional 3000 nouns, verbs, adjectives and adverbs were rated. These were selected to include everyday words which had been missed because of the random selection in the first round, as well as content-specific and academic vocabulary used in primary schools, which is in line with one of the goals of the MEGAHR project to develop direct vocabulary instruction in Croatian schools. Two sources were used: 1500 frequent everyday words were excerpted from the Croatian Frequency Dictionary (Moguš et al., 1999), and 1500 words were extracted from textbooks of Croatian, mathematics, history, geography and science used in primary school grades 4, 5 and 6. All 6000 items were coded for word length (in characters), animacy, word class and raw frequency in hrWaC. Animacy was coded based on binary natural categories combined with a morphological criterion (also see Radanović et al., 2016). Plants (e.g. *hrast* “oak”) and groups of people (e.g. *razred* “class”) were marked as inanimate, whereas supernatural, anthropomorphic and dead entities as animate (e.g. *vrag* “devil”, *mrtvac* “corpse”), based on the differences in morphological marking of masculine nouns. Preliminary results of the first round of data collection, focusing on the dimensions of concreteness and imageability and testing the dual-coding theory, were published in Croatian (Peti-Stantić et al., 2018). In this paper, we present the complete database, i.e. the data for all the measured variables collected in both rounds of the data collection.

#### Instruments

The lists used in the two rating rounds were mixed with respect to word class, with random assignment of words from different word classes to each list. The 6000 items were distributed over 60 lists, each containing 100 words which were counterbalanced across list versions (A and B) to control for the method (order) effect. The participants rated two variables on the same sheet (concreteness and subjective frequency or imageability and AoA).

The instructions (see Appendix) were formulated to avoid the ambiguity resulting from the overlap between concreteness and imageability. Concreteness was defined as a category “in the (material) world”, underlining its perceptive component (“concrete words stand for something that can be directly experienced through one’s senses and actions”) and contrasting it with abstractness (“abstract words refer to something which cannot be directly experienced through one’s senses and actions”). In contrast, imageability was defined as a category “in the mind”, i.e. the availability of mental imagery, explicitly including its various types (visual, auditory, olfactory, etc.). Both sets of instructions were modified from those previously used by Brysbaert et al. (2014b) for concreteness. Calibrator words were used only for the extreme points of the Likert-type scale (1–5) for each word class (e.g. concreteness: *tuljan* “seal”, *plivati* “to swim”, *slan* “salty”; abstractness: *pravda* “justice”, *morati* “to have to”, *poetski* “poetic”; high imageability: *kuća* “house”, *grmjeti* “to thunder”; low imageability: *nedosljedan* “inconsistent”, *smjeti* “be allowed to”, *aspekt* “aspect”).

Subjective frequency was operationalized using a Likert scale, where the participants were asked to report how frequently they encounter a word: almost never (1), once a year (2), once a month (3), once a week or (4) once/several times a day (5). AoA was defined as “understanding the meaning of a word at a certain age”, so the participants were asked to estimate the age at which they could say they knew the meaning of a word. Such a continuous measure of AoA correlates highly (above .80) with more frequently used Likert-like rating-scale measures, where the participants have to mark a number indicating an age range (defined in advance by researchers) in which they acquired a word (Kuperman et al., 2012). We decided to use the continuous measure of AoA because it has been shown to have several advantages compared to rating-scale measures: (1) the participants find it easier to comprehend, (2) it provides more precise information and overcomes the problem of a restricted response range immanent to rating-scale measures, and (3) it enables the calculation of additional variables, such as the number of years the word is known (Ferrand et al., 2008; Ghyselinck et al., 2000; Kuperman et al., 2012).

The instructions were printed on the first page of each questionnaire sheet, together with a brief set of sociodemographic questions on the participants’ age, sex, place of birth, city of residence, knowledge of foreign languages and hours spent reading every day.

#### Procedure

The entire MEGAHR project and all its research procedures were approved by the Research Ethics Committee of the University of Zagreb Faculty of Humanities and Social Sciences, and participation in the study was voluntary and anonymous. The questionnaire was administered using the traditional pen-and-paper method during regular classes. No specific time limit was set, but the participants usually took 20 minutes to rate the 100 words on the list that was assigned to them. Missing values, which were the result of the participants accidentally or intentionally skipping a word, were not supplemented by additional ratings.

### Results

The CPD, which includes all the results for all the 6000 words, is freely available at 10.17234/megahr.2019.hpb. The results include the mean, median, standard deviation, minimum and maximum values and the number of raters (separately for male and female raters, and as a total value) for the psycholinguistic variables (concreteness, imageability, subjective frequency and AoA). Moreover, linguistic characteristics of each word are also coded, i.e. word length in number of characters, word class (noun, verb, adjective and adverb), animacy and gender for nouns and raw frequency in the hrWaC corpus. Currently, the rated words appear only in Croatian, but English translations will be provided by the end of the project.

#### Reliability and validity

The reliability of the ratings for concreteness, imageability, AoA and subjective frequency was calculated by randomly dividing the participants into two subgroups of equal size and computing the correlation between averaged estimation by item. The reliability indexes were calculated on 5000 different randomizations of the participants. The obtained split-half correlations ranged from .86 to .90 (*M* = .88) for concreteness, from .81 to .88 (*M* = .85) for imageability, from .89 to .93 (*M* = .91) for AoA, and from .87 to .90 (*M* = .89) for subjective frequency. Moreover, there were no significant correlations between the place on the list and ratings for any of the variables, except for imageability ratings on list A and AoA ratings on list B which were both very small at .04 (*p* < .01). This corroborates the reliability and validity of the collected ratings.

Our data also correlate with two published databases for Croatian. The correlations for imageability and subjective frequency between our database and Kuvač Kraljević and Olujić (2018) for 266 and 264 words, respectively, shared between the two databases was high (*r* = .86, *p* < .01 for imageability and *r* = .83, *p* < .01 for subjective frequency). The correlation for concreteness with Ćoso et al. (2019) for 1123 shared words was *r* = .71, *p* < .01, which is rather high, given the different instructions provided to the participants.

#### Descriptive statistics and the impact of word class

Table 2 contains descriptives for all variables in the database, separately for each word class as well as for all 6000 words. To test whether psycholinguistic and linguistic word features differ for nouns, verbs, adjectives and adverbs, we performed one-way ANOVAs with word class as an independent variable. We did not test for differences in the objective frequency between word classes because not all words in the database were randomly selected, as described in the Materials section. Therefore, differences in the objective frequency of the sample might not reflect real differences in the corpus.

**Table 2:** Descriptive statistics for 6000 words from the CPD

Variable	Word type	N	M	SD	Min.	Max.	1st Quartile	Median	3rd Quartile
Concreteness	Nouns	2617	3.62	0.85	1.27	5.00	2.97	3.63	4.37
	Verbs	1571	3.21	0.71	1.43	4.86	2.67	3.17	3.73
	Adjectives	1554	2.92	0.69	1.17	4.83	2.43	2.83	3.40
	Adverbs	258	2.66	0.64	1.33	4.43	2.20	2.59	3.13
	Total	6000	3.29	0.83	1.17	5.00	2.66	3.23	3.90
Imageability	Nouns	2617	3.84	0.77	1.29	5.00	3.27	3.90	4.53
	Verbs	1571	3.58	0.67	1.70	4.97	3.07	3.60	4.10
	Adjectives	1554	3.37	0.69	1.24	4.97	2.87	3.33	3.87
	Adverbs	258	3.09	0.66	1.76	4.77	2.63	2.97	3.60
	Total	6000	3.62	0.75	1.24	5.00	3.04	3.63	4.23
Age of acquisition	Nouns	2617	8.36	2.53	2.24	16.72	6.37	8.30	10.21
	Verbs	1571	7.80	2.16	3.00	14.43	6.10	7.67	9.35
	Adjectives	1554	8.84	2.23	3.70	15.27	7.23	8.86	10.40
	Adverbs	258	7.46	2.45	3.50	14.70	5.47	6.92	9.42
	Total	6000	8.30	2.39	2.24	16.72	6.40	8.27	10.03
Subjective frequency	Nouns	2617	3.24	0.78	1.14	4.97	2.67	3.23	3.83
	Verbs	1571	3.46	0.69	1.17	5.00	3.00	3.47	3.99
	Adjectives	1554	3.18	0.69	1.30	4.90	2.70	3.17	3.67
	Adverbs	258	3.91	0.81	1.30	5.00	3.50	4.10	4.52
	Total	6000	3.31	0.75	1.14	5.00	2.77	3.32	3.87
Frequency	Nouns	2617	66,077.88	162,126.28	2.00	4.075e +6	4927.00	15,532.00	62,690.00
	Verbs	1571	65,243.29	254,388.35	11.00	5.663e +6	4756.00	11,934.00	46,015.00
	Adjectives	1554	49,090.40	172,019.94	1.00	3.582e +6	4473.25	10,461.50	35,283.00
	Adverbs	258	215,711.79	413,298.93	4.00	3.077e +6	16,827.50	71,288.00	223,390.75
	Total	6000	67,893.86	210,739.43	1.00	5.663e +6	4804.50	13,495.50	54,260.75
Length	Nouns	2617	7.23	2.46	2.00	18.00	5.00	7.00	9.00
	Verbs	1571	8.74	2.07	3.00	18.00	7.00	9.00	10.00
	Adjectives	1554	8.23	2.35	3.00	16.00	7.00	8.00	10.00
	Adverbs	258	6.58	2.05	2.000	12.00	5.00	6.00	8.00
	Total	6000	7.86	2.42	2.000	18.00	6.00	8.00	9.00

Results of ANOVAs revealed significant differences between word classes in all the psycholinguistic word features, as well as in word length (*Fs* were 346.33, 197.36, 61.30, 101.78 and 180.03, for concreteness, imageability, AoA, subjective frequency and word length, respectively; for all analyses *p* < .001, and *df* = 3, 5996; effect sizes: *η*^2^ = 0.15 for concreteness, *η*^2^ = 0.09 for imageability, *η*^2^ = 0.03 for AoA, *η*^2^ = 0.05 for subjective frequency and *η*^2^ = 0.08 for word length).

Post hoc Bonferroni tests revealed a statistically significant difference (*p* < .001) in all pairs of word classes in mean concreteness ratings, with nouns being rated as the most concrete, followed by verbs (nouns – verbs Cohen’s *d* = 0.51), adjectives (verbs – adjectives Cohen’s *d* = 0.42), and finally, adverbs as the most abstract words (adjectives – adverbs Cohen’s *d* = 0.38). The same pattern was observed for imageability, with nouns rated as the most imageable, followed by verbs (nouns – verbs Cohen’s *d* = 0.36), adjectives (verbs – adjectives Cohen’s *d* = 0.31), and finally, adverbs as the least imageable words (adjectives – adverbs Cohen’s *d* = 0.41).

As for subjective frequency and AoA, a Bonferroni test indicated that all pairs of word classes differed statistically significantly (*p* < .001) in subjective frequency except for the difference between nouns and adjectives (*p* = .052). Adverbs were rated as the most frequent, followed by verbs (adverbs – verbs Cohen’s *d* = 0.65), and nouns and adjectives as the least frequent (verbs – nouns Cohen’s *d* = 0.29). The same pattern was observed for the AoA. The Bonferroni test indicated a statistically significant difference (*p* < .001) in all pairs of word classes, except for the difference between adverbs and verbs (*p* = .205). Adverbs and verbs were rated as the earliest acquired words, followed by nouns (verbs – nouns Cohen’s *d* = 0.23), and adjectives as acquired the latest (nouns – adjectives Cohen’s *d* = 0.20) (see Table 2 for means). Post hoc Bonferroni tests revealed that there is a difference in length between all pairs of word classes (*p* < .001). The shortest word class—adverbs—were shorter than nouns (Cohen’s *d* = 0.27), nouns were shorter than adjectives (Cohen’s *d* = 0.41), and finally, adjectives were shorter than the longest word class—verbs (Cohen’s *d* = 0.23). We report effect sizes for the smallest differences obtained in the post hoc tests (all others were larger).

#### Relations between psycholinguistic and linguistic word features

**Fig. 1:**
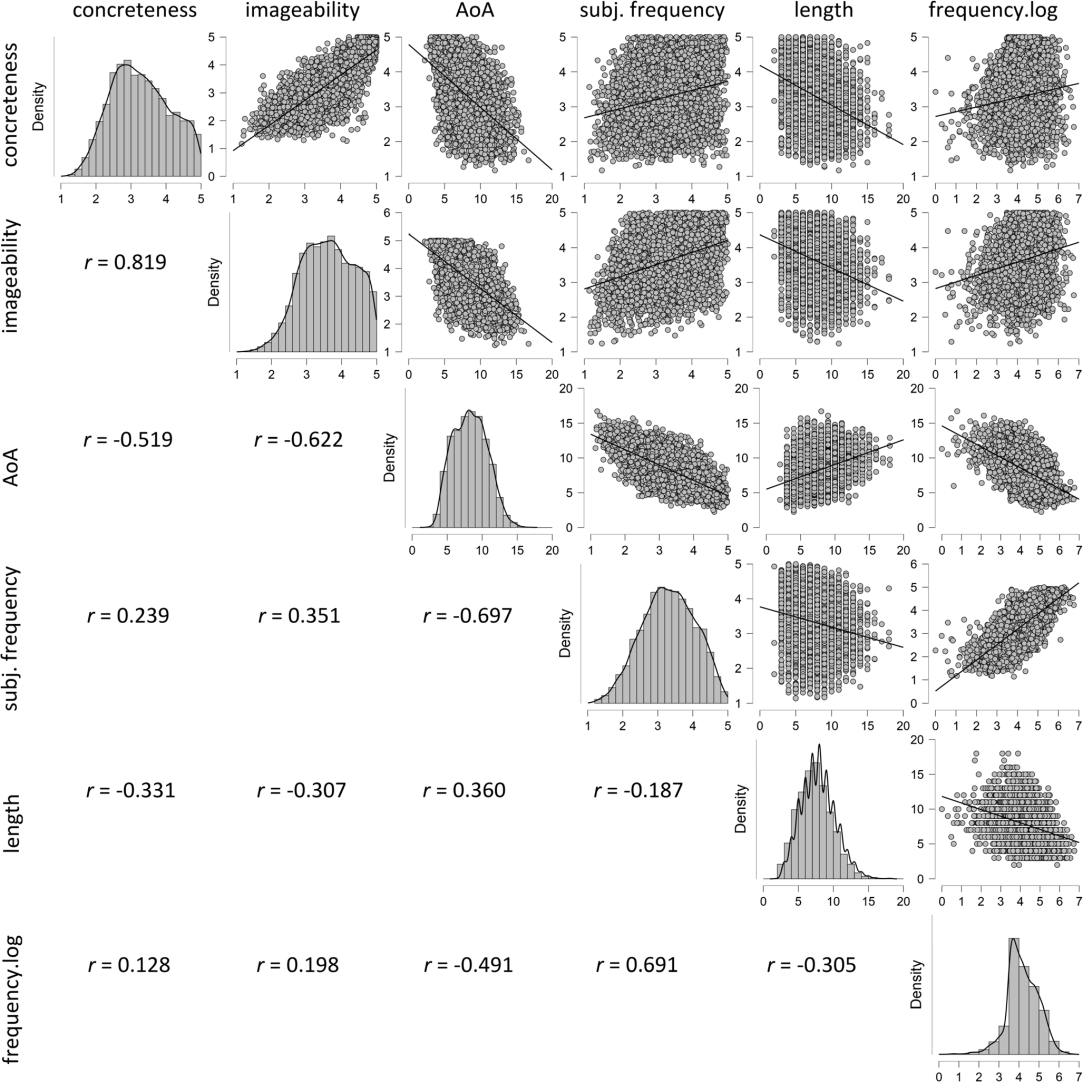
Correlations between psycholinguistic and linguistic features of words in the CPD

Pearson correlations between all word features included in the CPD, calculated for all 6000 words, are presented in Fig. 1. Because the objective frequency variable is extremely skewed, it was log-transformed prior to the calculation of the correlation coefficients. All correlations were statistically significant at *p* < .001, although their magnitudes differed considerably. Expectedly, the highest correlation was obtained between concreteness and imageability, indicating that words rated as more concrete also tended to be rated as more imageable, e.g. *odijelo* “suit” (Conc 4.7, Imag 4.8), *novčić* “coin” (Conc 5, Imag 4.9), *normalizacija* “normalization” (Conc 1.6, Imag 1.9) and *neshvatljiv* “incomprehensible” (Conc 1.8, Imag 1.8). However, given the .819 correlation, the CPD also contains words that diverge from this pattern exhibiting high concreteness and low imageability, e.g. *primatelj* “recipient” (Conc 4.2, Imag 2.8), *kositar* “tin” (Conc 4.2, Imag 2.96), and low concreteness and high imageability, e.g. *mišljenje* “opinion” (Conc 1.2, Imag 4.2), *obećanje* “promise” (Conc 1.7, Imag 4.4). Reflecting the high association between imageability and concreteness, the pattern of relations of the two variables with other word features was similar. Thus, words rated as more concrete and more imageable tended to be estimated as acquired at a younger age, as being subjectively and objectively more frequent, and as being shorter. AoA had the highest correlation with subjective frequency, suggesting that words reported to be acquired at a younger age also tended to be rated as more subjectively frequent. Although a lower correlation was obtained between AoA and objective frequency, it reveals that words estimated as being acquired at a younger age are objectively more frequent than those reported to be acquired at an older age. Furthermore, words rated as being acquired at a younger age tended to be shorter than those rated as being acquired at an older age. In addition to the mentioned correlations between subjective frequency and the psycholinguistic variables, subjective frequency had a high positive correlation with objective frequency, and a low negative correlation with word length. Finally, the two linguistic word features correlated moderately negatively: words which are objectively more frequent tend to be shorter. Pearson correlations calculated separately in the subsamples of the four word classes showed that the overall pattern and the magnitude of associations between the word features were very similar across the four word classes.

## Interim discussion

The values of all word features are comparable to values available in databases for other languages cited earlier. AoA means in our study may seem somewhat higher than those obtained in other studies using a continuous AoA measure for French (Ferrand et al., 2008), Italian (Montefinese et al., 2019) and Dutch (Moors et al., 2013). However, given that AoA correlates with word length across languages (shorter words tend to be acquired at younger ages), the differences may, in part, be ascribed to word length. In fact, all the studies in question normed words with a shorter average length than our study. Another reason for differences in AoA may be age of participants, with older participants tending to give higher ratings than younger ones (Kuperman et al., 2012). This is the case with Kuperman et al. (2012), who reports the highest AoA of all the studies cited in this paragraph, probably because 45% of the participants were older than 30 (whereas ratings in all the other studies were collected from young adults, mostly university students, in their early twenties).

All the correlations obtained in the data follow the expected pattern found in previous studies and databases (e.g. Altarriba et al., 1999; Bird et al., 2001; Cameirão & Vicente, 2010; Desrochers & Thompson, 2009; Montefinese et al., 2019; Paivio et al., 1968; Soares et al., 2017; Stadthagen-Gonzalez & Davis, 2006), whereby words rated as more concrete are generally likely to be more highly imageable, shorter, acquired earlier and having a higher subjective frequency rating. What is perhaps more surprising is the order of the reported AoA and subjective frequency, starting with adverbs and verbs, followed by nouns, and ending with adjectives. To our knowledge, reports of rated AoA and word class interactions are infrequent in the literature. In a recent study on Italian (Montefinese et al., 2019), the order of mean AoA for verbs, nouns and adjectives was the same as the one obtained in the present study. A study for Portuguese (Cameirão & Vicente, 2010) reports adverbs being acquired earliest. They explained this by the higher presence of earlier acquired words such as “only” and “now” in their dataset. The same seems to be true of our data, because the 258 adverbs were selected in the second round, so as to reflect highly frequent and everyday vocabulary, rather than a random sample from a wide frequency range of items. This resulted in the selection of more basic adverbs, such as *sutra* “tomorrow”, *dobro* “well”, *tiho* “quietly”, *još* “more”, *gore* “up”, *tu* “here”, *koliko* “how much”, etc., with more complex adverbs missing from the database. This is also related to the subjective frequency rating, which is highest for adverbs, and corresponds to their highest raw frequency in the corpus, as well as their lowest length in letters. ANOVA with word length, subjective frequency and log-transformed objective frequency as covariates showed that the significant differences between word classes in AoA remain after controlling for length and frequency (*F* = 58.31; *p* < .001), with all post hoc differences significant (*p <* .001), except for the difference between nouns and adjectives. This indicates that an explanation based on word class-related factors may be worth exploring, although the overall effect size of word class on AoA is small.

Moreover, adverbs of time and space are clearly deictic in nature, which means that they more readily relate to the immediate environment. The result whereby verbs were rated as being learned earlier than nouns clashes with Gentner’s natural partition hypothesis (Gentner, 1982), which claims that nouns are conceptually more basic than verbs because they refer to people and things rather than relations. Our result gives more credence to accounts which refer to other language-related and sociocultural factors as more significant (Stoll et al., 2012).

The differences in concreteness and imageability between word classes partially correspond to similar findings in other languages. Thus, Bird et al. (2001) found differences between nouns and verbs for English, with verbs being significantly less imageable than homonymous nouns. Similarly, significant differences in imageability ratings were found between Norwegian nouns, verbs and adjectives (Simonsen et al., 2013), with the same noun > verb > adjective cline as found in the present analysis. To our knowledge, differences between concreteness and word class, as well as the position of adverbs in the cline, have not been tested. We will return to these issues in the general discussion.

## Study 2: Extrapolations of concreteness and imageability

The aim of the second study was to investigate (1) to which extent we can extrapolate concreteness and imageability ratings in our lexicon of 6000 entries to the remainder of the Croatian lexicon and (2) to extend the existing body of research on extrapolation of psycholinguistic variables by focusing on (a) variation between word classes and (b) the interaction between concreteness and imageability.

### Method

Extrapolations in this work were performed by using word embeddings as explanatory variables and one of the two variables, concreteness or imageability, as response variable. In line with Ljubešić et al. (2018), we used pretrained fastText embeddings. fastText is a Python and C++ library for learning word representations (vectors, embeddings) and text classification (Bojanowski et al., 2017), but it also offers pretrained word vectors for 157 languages, trained on huge amounts of text from Wikipedia and the Common Crawl for the respective languages (Grave et al., 2018). To perform machine learning on the dataset, we used the support-vector machine model with a radial basis kernel, a setup which proved optimal in our previous research dealing with Croatian and extending the ratings to 77 other languages (Ljubešić et al., 2018). To evaluate the results of our machine learning experiments, we performed fivefold cross-validation, i.e. we split our data in five folds, and performed model training over four folds and model evaluation on the fifth left-out fold, performing this procedure in five iterations. With such an approach, we ensure best estimates of the model performance as all available data was used at some point for model evaluation. Spearman correlations were used for evaluation, as (1) it is more resistant to outliers and (2) our response variable is probably closer to an ordinal than an interval variable.

Different to our previous work (Ljubešić et al., 2018), we perform these experiments on the full MEGAHR database extrapolating our results to the remaining 100,000 words of the Croatian vocabulary, we compare the results with the extrapolations done on the MRC database (Wilson, 1988) and the BWK database (Brysbaert et al., 2014b) in order to ensure comparability, and we additionally investigate whether the quality of the predictions depends on the word class of the words for which we performed the predictions.

### Results and discussion

The results show that both concreteness and imageability are highly predictive from word embeddings within Croatian and English. The Spearman’s correlation coefficients for concreteness were *r*_s_ = .76 (MEGAHR), *r*_s_ =.87 (MRC) and *r*_s_ = .89 (BWK), whereas they were *r*_s_ = .68 (MEGAHR) and *r*_s_ = .80 (MRC) for imageability. A larger dataset seems to facilitate machine learning, which is why English extrapolations are better than Croatian. The correlation for concreteness for the MRC database for our model is slightly better than the one published by Hollis et al. (2017), which is at *r* = .84. We are not aware of any other extrapolated values for imageability.

Concreteness predictions correlate with human predictions better than predictions for imageability, and the difference between them is significant, both in the case of MEGAHR (*z* = 9.28, *p* < .01), and in the case of MRC (*z* = 10.56, *p* < .01). Differences in predicting concreteness and imageability between MEGAHR and MRC are also significant (*z* = −16.86, *p* < .01; *z* = −4.5, *p* < .01).

We performed a separate evaluation of the models trained and evaluated on the MEGAHR dataset, by separating the predictions by word class. The results show that the overall correlations for concreteness for nouns are stronger than those for verbs, adjectives and adverbs (*r* = .77, *r* = .70, *r* = .62, *r* = .48, respectively) with a clear cline between them. The results for imageability show the strongest correlation for nouns (*r* = .68), and weaker correlations for the other word classes (*r* = .62 for verbs, *r* = .62 for adjectives and *r* = .55 for adverbs).

Finally, we compared concreteness and imageability extrapolations with human ratings for all the word classes. The results show that the overall correlations between concreteness and imageability are higher in machine predictions (*r* = .93) than they are in human ratings (*r* = .82). We then compared the direction of the predictions for a set of 5301 nouns, verbs, adjectives and adverbs. The sample was obtained by removing the training set and manually discarding any obvious errors in the predicted data (e.g. predicted values beyond the expected range of 1–5). We subtracted the predicted concreteness or imageability mean ratings from the human-rated concreteness or imageability mean ratings separately for each variable, obtaining a score that shows the direction of the prediction. Negative scores indicate that the mean of the predicted value is greater than the mean of the human rating; i.e. in the case of negative scores, extrapolated values are overpredicted in relation to human ratings. Positive scores represent underprediction by machine learning. We performed an ANOVA on the means to test for word class differences.

The results for concreteness showed a significant difference between word classes, with a small effect size (*F* = 32.74, *p* < .001, *df* = 3, *η*^2^ = 0.02). Post hoc Bonferroni tests revealed that all pairs of word classes except for nouns and verbs differed statistically significantly (*p* < .001), with nouns being consistently underpredicted, and adjectives and adverbs consistently overpredicted (nouns – adjectives Cohen’s *d* = 0.25; adjectives – adverbs Cohen’s *d* = 0.26 and verbs – adjectives Cohen’s *d* = 0.17). The results for imageability also showed a significant difference between word classes, but the effect size was minimal (*F* = 15.28, *p* < .001, *df* = 3, *η*^2^ = 0.01).

Concreteness was easier to predict than imageability, which may mean that concreteness—i.e. availability to the senses—is captured more easily using distributional data. Notionally, concreteness is verifiable by our senses; it refers to entities that are present in our environment and is textually related to a number of lexical items (something concrete may be seen, heard, smelled, touched, etc.). In contrast, imageability is an “internal” (“one’s minds’ eye”) capacity of a human being to invoke a mental image. The mental image is based on the knowledge of an external stimulus; however, invoking a mental image makes sense only if the stimulus is not present (you do not need to imagine a desk or a foul smell when you can see it or smell it). Imageability is not as clearly related to a number of basic and readily available lexical items (only *imagine* comes to mind).

Therefore, because of its correlation with concreteness, imageability may depend on distributional evidence only inasmuch as it corresponds to concreteness (in the typical high concreteness – high imageability and low concreteness – low imageability cases). In the remaining cases, distributional evidence may not be sufficient. This is evident from a comparison of predictions of concreteness and imageability for words that participants rated as abstract and highly imageable. A total of 98 such words, with concreteness rated below 2.5 and imageability above 3.5, were found in our set (some of them mentioned in the first study). Among them, imageability was predicted within .5 of the mean of the human rating in four cases, and in 94 cases going beyond this. In contrast, in the same set, concreteness was predicted within .5 of the mean of the human rating in 59 cases, with the remaining 39 cases going beyond the arbitrary .5 limit. This suggests that distributional evidence works better for concreteness than for imageability. This is in line with Crossley et al.’s (2013) study where textual categories such as hypernymy and lexical diversity had a significant correlation with concreteness but not with imageability (Crossley et al., 2013, p. 152). The correlation of this evidence with emotional grounding (Kousta et al., 2011) or the mode of acquisition (Della Rosa et al., 2010) still remains to be tested.

## General discussion

The results of the human rating study showed expected correlations between psycholinguistic variables, as well as an effect of word class on the psycholinguistic variables of AoA, subjective frequency, concreteness and imageability. For concreteness and imageability, there was a cline nouns > verbs > adjectives > adverbs. In the machine learning study, we focused on concreteness and imageability and obtained results which, in the whole sample, correlated with human predictions. However, the size of the correlations across word classes followed the same cline. Finally, a similar cline was obtained for the direction of the predictions for concreteness. The system underpredicted the concreteness of nouns, and overpredicted the concreteness of adjectives and adverbs. Imageability predictions followed suit, but the effect size was minimal.

These results are in line with the conceptual definition of word classes given in the introduction, which suggests that word class should be taken as a shorthand for semantic and distributional characteristics, at least within a single language. Simply put, nouns typically denote objects, verbs denote relations between objects, and adjectives and adverbs denote qualities and external characteristics. There is a clear conceptual decrease in concreteness and imageability: nouns are most highly concrete and imageable because they are conceptually related to entities that are less diffuse and have a spatial basis, and the decrease in the spatial basis of verbs, adjectives and adverbs, and their increasingly relational nature leads to lower concreteness and imageability. In other words, word class adds a schematic conceptual frame to the lexical meaning of words.

This is also visible from the differences in the direction of the prediction for concreteness in the machine learning experiment, where this “conceptual frame” seems to be more significant for humans than when using machine learning based on collocational evidence. It seems that, at least for some nouns, humans give more weight to the conceptual factor of “nouniness” (i.e. thing-like representation) rather than to distributional evidence. By the same token, people see some adjectives and adverbs as more diffuse and hence less concrete, perhaps because adjectives and adverbs require conceptual separation of the quality from the object it describes. In contrast, relying on collocational evidence (where no such separation occurs), the system predicts them as more concrete than humans.

Regarding the theoretical significance of word class for the context availability theory, the dual-coding theory and grounding theories, it seems that all of them should incorporate grammatical information as part of their program. It seems that various flavors of grounding theories (e.g. the words-as-social tools theories; Borghi & Binkofski, 2014) are best suited for a principled step in this direction. This is also suggested in a reaction time study (Scorolli et al., 2011), which alongside concreteness and imageability, takes word class into account.

The fact that imageability was less dependent on distributional evidence provides additional insight into Mandera et al.’s (2015) warning that machine learning models may introduce semantic artifacts that do not appear in human ratings. Our work suggests that extrapolations diverge from human intuitions when distributional evidence is insufficient to capture conceptual characteristics. Viewed from the perspective of grounding theories, this is not surprising, because distributional evidence lacks direct conceptual, social and affective grounding crucial in any human learning and communication. In other words, unless such evidence is somehow included, machine learning models are likely to produce artifacts. Similar general points have recently been made in the natural language processing community (Bender & Koller, 2020; Bisk et al., 2020).

The notion of World Scope (Bisk et al., 2020) as a way to conceive of the progress of natural language processing is illuminating in this sense. In essence, World Scope describes evidence considered in natural language processing: written, perceptual, embodied (interactive) and social (interpersonal). Distributional evidence used in extrapolations presented and quoted in this paper belongs to the World Scope of the written world. Bringing in perceptual grounding has—to some extent—been done in the visually supervised language model (Tan & Bansal, 2020), with work on interactive and interpersonal grounding largely still to follow (Bisk et al., 2020, p. 8721–8725).

Textual evidence from the written world works as well as it does because it is an indirect, but vast, representation of the human experience, which includes the experience of the perceptual, embodied, and social world. We have reached a point where the written world includes sufficient information to be able to provide a convincing representation. However, it is a representation, rather than being directly grounded like human experience. It seems that imageability may require grounding via additional, quite probably visual, stimuli to be modeled (more) convincingly, so that the artifacts are not a result of using only one type of evidence (and the wrong kind of evidence at that). Characteristics other than imageability that require more than just textual evidence seem to include emotional valence, arousal and dominance (Hollis et al., 2017; but see Buechel et al., 2020).

## Conclusions and further research

The CPD provides ratings of concreteness, imageability, AoA and subjective frequency for 6000 Croatian nouns, verbs, adjectives and adverbs. These ratings are coupled with data on word length in letters, word class, animacy and corpus frequency. The observed correlations indicate a correspondence between the data collected in the database and the relevant literature. Nouns, verbs, adjectives and adverbs exhibit differences in imageability and concreteness, which were related to their conceptual content and grammatical valence. Although no definitive conclusions on the differences between word classes in AoA and subjective frequency could be reached, the results are not inconsistent with the provided word class-based conceptual explanations.

Using computational modeling techniques, concreteness and imageability ratings were extended to the remaining part of the Croatian lexicon not covered by the CPD. Concreteness was easier to extrapolate than imageability, and the same cline of word class differences was obtained for concreteness, with nouns being underpredicted and adjectives and adverbs overpredicted. This suggests, alongside other work such as Vigliocco et al. (2011), that grammatical differences, including word class differences, should be theoretically explored.

Several avenues of research remain. Work is underway to supplement the CPD by ratings of affective variables (valence and arousal). Given the role of word class in this work, and the relation between affective variables and concreteness and imageability (Kousta et al., 2011), it would be interesting to see whether affective variables vary with regard to word class. This may lead to further theoretical refinements, as well as a better understanding of the existence and nature of semantic artifacts that computational models introduce. Moreover, given the constructional view of language, the way in which psycholinguistic and affective variables are related to contextual factors should be explored, for instance, by looking into the facilitatory role of the context in acquiring and processing abstract words, as well as the correlation between AoA, concreteness and imageability, especially in connection with the development of reading proficiency and the capacity for deep reading (for striking differences between younger and older children, see Altarriba et al., 1999; Caramelli et al., 2004; Schwanenflugel et al., 1992).

## Appendices

**Table 1:** Number of Raters for Each Psycholinguistic Category

	Total				Female		Male	
	*M*	*SD*	*Min*	*Max*	*M*	*SD*	*M*	*SD*
Concreteness	30.20	1.35	20	36	21.98	3.83	6.90	3.39
Imageability	30.05	0.86	22	35	23.06	3.82	5.43	2.95
Age of acquisition	29.72	1.21	17	34	22.80	3.74	5.40	2.93
Subjective frequency	30.16	1.34	19	36	21.94	3.83	6.89	3.40

**ORIGINAL INSTRUCTIONS AND ENGLISH TRANSLATION**

**CONCRETENESS AND SUBJECTIVE FREQUENCY - CROATIAN**

Neke se riječi odnose na pojave iz stvarnosti koje se mogu neposredno osjetilno doživjeti. Kažemo da su značenja tih riječi **konkretna**. Do značenja drugih riječi ne može se neposredno osjetilno doprijeti. Značenja tih riječi obično definiramo drugim riječima. Kažemo da su značenja tih riječi **apstraktna**. Neke se riječi nalaze između tih dviju krajnosti.

Koristeći ljestvicu 1-5 označite u kojoj je mjeri **za Vas osobno** značenje riječi konkretno.

Konkretne će riječi biti ocijenjene višim ocjenama. Takve se riječi odnose na nešto što se može izravno doživjeti vlastitim osjetilima i djelovanjima. Najjednostavniji način objašnjavanja konkretnih riječi je pokazivanje (za objašnjenje riječi 'tuljan' možete pokazati tuljana ili sliku tuljana, za objašnjenje riječi 'plivati' možete jednostavno zaplivati ili pokazati nekome isječak iz filma u kojem netko pliva, za objašnjenje riječi 'slan' možete nekome dati da pojede malo soli).

Apstraktne će riječi biti ocijenjene nižim ocjenama. Takve se riječi odnose na nešto što se ne može izravno doživjeti vlastitim osjetilima ili djelovanjem, a značenje im se najčešće objašnjava drugim riječima. Nema jednostavnog načina pokazivanja značenja riječi 'pravda', no do njezina značenja možemo doći služeći se drugim riječima.

Nemojte razmišljati o mnogim značenjima riječi, nego se oslonite na prvi dojam.

**Table:**

1 apstraktno	2	3	4	5 konkretno
pravda				tuljan
smisao				majica
morati				jesti
koncipirati				plivati
poetski				slan
slobodan				drven

Riječi se razlikuju i po tome koliko se često susrećemo s njima. S nekim se riječima susrećemo vrlo često, dok se s drugima susrećemo iznimno rijetko. Cilj ovoga istraživanja je i procjena **subjektivne čestoće riječi**. Molimo Vas procijenite riječi koje slijede na ljestvici od 1 do 5.

S riječi za koju me se pita susrećem se: 1 = gotovo nikada, 2 = jednom godišnje, 3 = jednom mjesečno, 4 = jednom tjedno, 5 = jednom ili više puta dnevno.

**Table:**

1 Gotovo nikada	2	3	4	5 Jednom ili više puta dnevno

Istraživanje se provodi za potrebe istraživačkog projekta Hrvatske zaklade za znanost **„Modeliranje mentalne gramatike hrvatskoga: ograničenja informacijske strukture”.**

Podaci će biti korišteni u skladu s etičkim pravilima i zahtjevom za anonimnošću.

Koliko godina imate? __________Spol: **M / Ž**

Gdje ste rođeni? _________________________________

U kojem ste gradu proživjeli najveći dio života? ________

Kojim se stranim jezicima služite? ___________________

Koliko sati na dan prosječno provedete čitajući?

manje od 1 1-2 3-4 5-6 više od 6

**CONCRETENESS AND SUBJECTIVE FREQUENCY – ENGLISH TRANSLATION**

Some words refer to entities in the world which can be directly experienced through the senses. Meanings of such words are referred to as **concrete**. Meanings of other words refer to entities which cannot be directly experienced through the senses. They are usually defined by using other words. Meanings of such words are referred to as **abstract.** Some words are somewhere in between these two extremes.

Using a 1 to 5 scale, indicate **your own assessment** of how concrete the meaning of a particular word is.

Use higher scores for concrete words. Such words refer to entities which can be directly experienced through our senses and actions. The simplest way to explain what a concrete word means is by pointing or showing (to explain the word ‘seal’ you can point to a seal or a picture of a seal, to explain the word ‘swim’ you can simply start to swim or play a movie clip where someone is swimming, to explain the word ‘salty’ you can have someone taste a bit of salt).

Use lower scores for abstract words. Such words refer to entities which cannot be directly experienced through our senses or actions, and their meaning is most frequently explained by using other words. There is no simple way to point to or show the meaning of the word ‘justice’, but we can arrive at its meaning by using other words.

Do not think about the many different meanings a word may have, but rely on your first impression.

**Table:**

1 abstract	2	3	4	5 concrete
justice				seal
meaning				T-shirt
must				eat
conceptualize				swim
poetic				salty
free				wooden

Words also differ according to how frequently we encounter them. We encounter some words very frequently, whereas we encounter others very rarely. Another aim of this study is to collect **subjective frequency ratings**. Please, assess the following words on a scale from 1 to 5.

I encounter this word: 1 = almost never, 2 = once a year, 3 = once a month, 4 = once a week, 5 = once or several times a day.

**Table:**

1 Almost never	2	3	4	5 Once or several times a day

This study is being conducted within the Croatian Science Foundation research project **The Building Blocks of Croatian Mental Grammar: Constraints of Information Structure**.

The data will be treated in line with ethical principles, and as completely anonymous.

How old are you? __________Sex: **M / F**

Where were you born? ____________________________

The city/place of residence where you spent most of your life ___________________________

Which foreign languages do you speak/understand: ____________________________________

On average, how many hours a day do you spend reading?

less than 1 1-2 3-4 5-6 more than 6

**IMAGEABILITY AND AGE OF ACQUISITION – CROATIAN**

**PREDOČIVOST RIJEČI**

Riječi se razlikuju prema tome do koje mjere pobuđuju mentalne predodžbe, npr. predodžbu slike, zvuka ili drugog osjetilnog doživljaja. Kod nekih riječi mentalne predodžbe dolaze brzo i lako, dok se kod drugih riječi pojavljuju teže ili sporije. Primjerice, riječ „kuća” vjerojatno će kod vas lako i brzo pobuditi mentalnu predodžbu kuće; riječ „grmjeti” vjerojatno će brzo i lako pobuditi predodžbu odgovarajućeg zvuka. Za razliku od toga, riječi „nedosljedan”, „smjeti” i „aspekt” teže i sporije pobuđuju mentalne predodžbe ili ih uopće ne pobuđuju. Cilj je ovoga istraživanja procijeniti riječi prema tome koliko lako ili teško prizivaju mentalne predodžbe. Za riječi koje kod vas lako i brzo pobuđuju mentalnu predodžbu upisat ćete višu ocjenu, a za riječi koje kod vas teže i sporije pobuđuju mentalnu predodžbu ili je uopće ne pobuđuju upisat ćete nižu ocjenu.

Koristite se ljestvicom od 1-5.

**Table:**

1 - nisko predočivo	2	3	4	5 - visoko predočivo

Nemojte razmišljati o mnogim značenjima riječi, nego se oslonite na prvi dojam. Nema točnih i netočnih odgovora, važna nam je **vaša osobna procjena.**

**DOB USVAJANJA**

Riječi se razlikuju i po tome kada smo ih naučili. Neke smo riječi naučili vrlo rano, dok smo druge naučili kasnije tijekom školovanja i života. Cilj ovoga istraživanja je i procjena **dobi u kojoj mislimo da smo naučili neku riječ**. Naučiti neku riječ znači razumjeti njezino značenje u primjerenim kontekstima, čak i ako je sami ne upotrebljavate. Molimo Vas upišite **s koliko ste** **približno** **godina naučili pojedinu riječ**.

Istraživanje se provodi za potrebe istraživačkog projekta Hrvatske zaklade za znanost **„Modeliranje mentalne gramatike hrvatskoga: ograničenja informacijske strukture”.**

Podaci će biti korišteni u skladu s etičkim pravilima i zahtjevom za anonimnošću.

Koliko godina imate? __________Spol: **M / Ž**

Gdje ste rođeni? _________________________________

U kojem ste gradu proživjeli najveći dio života? ________

Kojim se stranim jezicima služite? ___________________

Koliko sati na dan prosječno provedete čitajući?

manje od 1 1-2 3-4 5-6 više od 6

**IMAGEABILITY AND AGE OF ACQUISITION – ENGLISH TRANSLATION**

**IMAGEABILITY**

Words differ in the extent to which they evoke mental images, for instance a mental visual image, an image of a sound or of another sensory experience. For some words, mental images come quickly and easily, whereas for others they appear with more difficulty or more slowly. For example, the word ‘house’ will probably evoke a mental image of a house in your mind easily and quickly; the word ‘to thunder’ will probably evoke the image of the corresponding sound quickly and easily. In contrast, words such as ‘inconsistent’, ‘be allowed to’, and ‘aspect’, will evoke mental images more slowly and with more difficulty, or will not evoke them at all.

The aim of this study is to assess words according to the ease or difficulty that they evoke mental images. Use higher scores for words which evoke mental images quickly and easily. Use lower scores for words which evoke images with more difficulty and more slowly, or do not evoke them at all.

Use a 1 to 5 scale.

**Table:**

1 - low imageability	2	3	4	5 - high imageability

Do not think about the many different meanings a word may have, but rely on your first impression. There are no correct or incorrect answers; what we need is **your personal judgement.**

**AGE OF ACQUISITION**

Words also differ according to when we learned them. We learned some words very early, whereas we learned others later, in school or our later life. The aim of this study is also to assess **the age at which we believe we learned a word**. To learn a word means to understand its meaning in appropriate contexts, even if we do not use the word ourselves. Please enter the **approximate** **age when you learned a certain word**.

This study is being conducted within the Croatian Science Foundation research project **The Building Blocks of Croatian Mental Grammar: Constraints of Information Structure**.

The data will be treated in line with ethical principles, and as completely anonymous.

How old are you? __________Sex: **M / F**

Where were you born? ____________________________

The city/place of residence where you spent most of your life ___________________________

On average, how many hours a day do you spend reading?

less than 1 1-2 3-4 5-6 more than 6
